# Metabolomics-Based Study of the Effect of Raw Materials to the End Product of Tempe—An Indonesian Fermented Soybean

**DOI:** 10.3390/metabo10090367

**Published:** 2020-09-11

**Authors:** Adinda Darwati Kadar, Made Astawan, Sastia Prama Putri, Eiichiro Fukusaki

**Affiliations:** 1 Department of Biotechnology, Graduate School of Engineering, Osaka University, 2-1 Yamadaoka, Suita, Osaka 565-0871, Japan; adinda_darwati_kadar@bio.eng.osaka-u.ac.jp (A.D.K.); fukusaki@bio.eng.osaka-u.ac.jp (E.F.); 2 Department of Food Science and Technology, Faculty of Agricultural Engineering and Technology, IPB University, Jl. Raya Dramaga, Bogor, Jawa Barat 16680, Indonesia; astawan@apps.ipb.ac.id

**Keywords:** food metabolomics, GC-MS, Indonesian tempe, starter culture, tempe

## Abstract

Tempe is a fermented soybean food from Indonesia, made by inoculating *Rhizopus* spp. onto cooked and dehulled soybean. Tempe has been a part of Indonesian culture since the 16th century and is now produced globally as a highly nutritious plant-based food. Despite a longstanding history on the production of tempe, very few studies have been reported to understand the effect of raw material to the end product metabolite composition. In this research, we applied GC/MS-based metabolite profiling to investigate the effect of various possible factors that might affect the final product (environmental factors, raw materials, and starter cultures). Representative samples were tempe produced by Indonesian industries, Japanese industries as well as laboratory made tempe. The results showed that both environmental factors and raw materials (soybean, water, and starter culture) contributed to the tempe metabolite profile. Here we found the possibility that starter cultures might play a greater role to determine the metabolite profiles compared to other tested factors. This research might provide useful insights for the larger scale industries to maintain the quality of tempe for the benefit of the consumers.

## 1. Introduction

Tempe, also well-known as “soybean cake”, is widely consumed in Indonesia and all over the world for its distinguished taste and nutritional values [1,2,3,4]. In Indonesia, tempe has been one of the most consumed protein sources for hundreds of years and known to be more preferable than meat and poultry [5]. It was reported that the fermentation process during tempe production improved the bioavailability of the protein, fiber, carbohydrates, isoflavones, vitamins [6,7,8]. These characteristics have made tempe a healthy protein source for plant-based diets, vegans, and vegetarians [9,10]. We argue that this is one of the reasons behind tempe popularity in many foreign countries (outside of Indonesia) as plant-based diets popularity has also been increasing in the past few decades. For example, middle-scale industries in Japan have been producing tempe and the products are readily available in the online marketplaces.

It is worth to note that tempe popularity does not parallel with the progression in the effort to understand the industrial fermentation of tempe scientifically. The available publications in tempe research provide detailed but fragmented knowledge about microbial studies during fermentation [11,12,13], dietary values [14,15], and health benefits [16,17] of tempe. Little research has focused on how to comprehensively understand the production processes of soybean tempe in a rather broader view.

**Figure 1:**
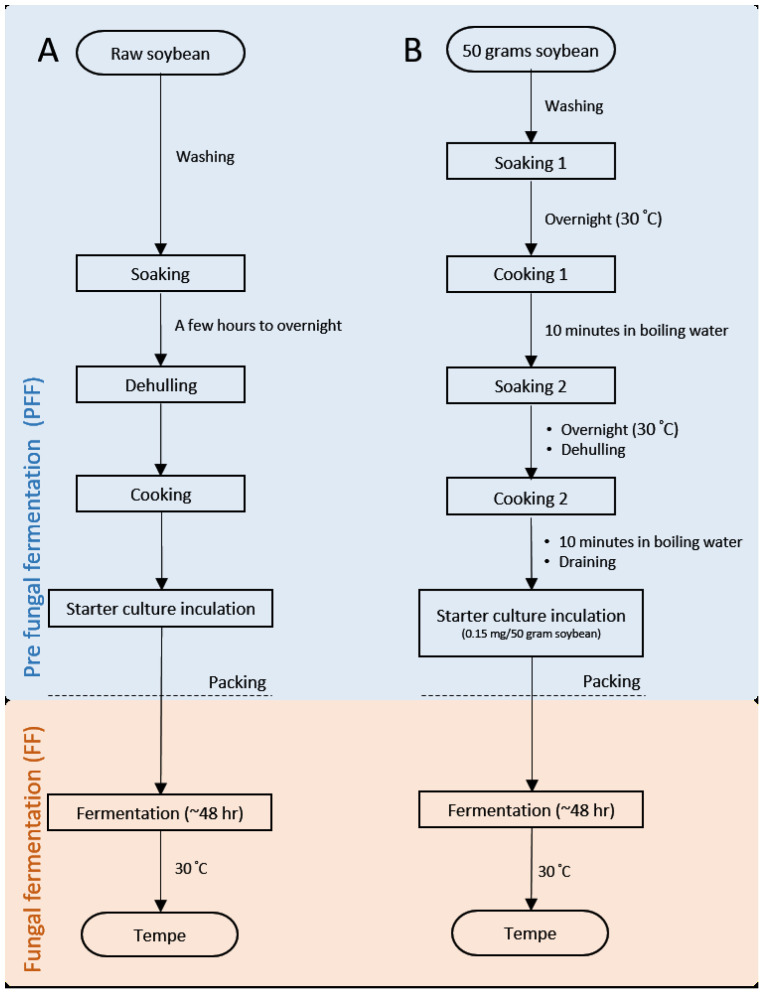
The workflow of tempe production process. (**A**) The general steps of tempe making procedure and (**B**) the methods of tempe making used in this research. The steps marked in blue area and orange area herein will be referred as to pre fungal fermentation stage (PFF) and fungal fermentation (FF) consecutively.

As tempe holds high cultural and economic values, it is important to obtain deeper understanding of tempe production in general. The step-by-step production of tempe in one industry is commonly not identical to the others. The technique will be heavily influenced by the common knowledge that is inherited within regions [18] and/or the practicality. However, there are general steps that are essential in tempe making, i.e., soaking of the raw soybeans, dehulling of the outer skin of soybean, cooking to soften the beans, culture starter inoculation, packing, and finally fungal fermentation. (Figure 1). In this article, the steps before packing will be referred to as pre fungal fermentation (PFF) while the step after as fungal fermentation (FF).

During PFF there are aspects that might influence the quality of the end products. For example, the soybean quality, water quality (used for soaking and cooking), and the quality of starter culture [19]. Cooking time and method might also give impact to the product. It was reported [11] that each step in the tempe production process affects the microbiome profiles in tempe. Some speculations were also made that each raw material contributed to the microbiome profiles of tempe, hence chemical composition of the product (pH, moisture, ash, fat, and carbohydrate).

Although the standard guidelines for tempe industry have been published locally in Indonesia (SNI 3144:2015) [20] and internationally (CODEX STAN 313R-2013) [21], direct comparison of how the differences in location and production processes will affect tempe product is still understudied. Better understanding of the effect of tempe production (i.e., fermentation conditions, raw materials, and production processes in general) to the end product of tempe will be beneficial to improve and to maintain tempe quality in the industries.

Metabolite profiling by means of metabolomics workflow has been applied to many fermented food products such as sourdough [22], douchi [23], kimchi [24], cheese [25], etc. Through applying a metabolomics approach to analyze the metabolite composition, the science behind the food processing has not been revealed. The findings from this research will be expected to be applied to improve the production for the benefit of both producers and the consumers.

Previous research [18] demonstrated the usefulness of untargeted metabolomics in the comparative study of tempe from different production regions and production processes. One of the important points raised from this research was that the environmental condition during fermentation in each region might contribute to the differences in metabolite profiles of tempe. It was proposed, although remains unclear, that the unique characteristics of tempe from different regions might have resulted from distinct development of the microbial communities during fermentation in the production region.

In the present study, we applied a metabolite profiling approach to a comparative study on tempe. From our previous study, possible factors that were considered influential in the final product metabolite composition were environmental factors, raw materials, and starter cultures. Here, we aim to propose a more comprehensive understanding of the effect of stated factors to tempe product through analyzing the metabolite profiles of laboratory-made tempe as well as industrial samples from different regions in Indonesia and Japan.

## 2. Results and Discussion

### 2.1. The Place of Production Affected Metabolite Profiles of Tempe More Than Fermentation Place

In this research we investigated the effect of environmental condition, soybean, water, and starter culture to the end product of tempe. To achieve this objective, we separated the experiment and discussion into three experiment sections.

The first experiment was called a cross fermentation experiment. This step aimed to investigate whether the environmental aspects during PFF or FF would give more impact to the metabolite profiles of tempe. Three industries in three different locations in Indonesia (Bogor, Ngawi, and Gunung Kidul) were involved. Metabolomics workflow was applied to the samples according to the previously published method [18].

**Figure 2:**
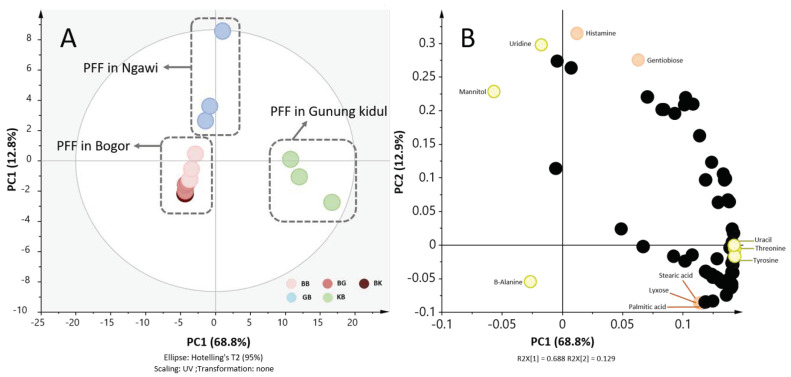
Principal component analysis (PCA) score and loading plot of cross fermentation experiments. Seventy-one annotated metabolites, n = 3. (**A**) PCA score plot shows separation between tempe in which PFF happened in Bogor, Ngawi, and Gunung Kidul along the PC1 (explain 68.9% of the variance), BB = PFF in Bogor, FF in Bogor; BK = PFF in Bogor, FF in Gunung Kidul; BG = PFF in Bogor, FF in Ngawi; GB = PFF in Ngawi, FF in Bogor; KB = PFF in Gunung Kidul, FF in Bogor. (**B**) PCA loading plot. Three representative metabolites contributing to the separation were mannitol, β-alanine, uridine, uracil, threonine, and tyrosine for PC1 and histamine, uridine, gentiobiose, stearic acid, lyxose, and palmitic acid for PC2.

Seventy-one metabolites were annotated from GC/MS analysis and were subjected to principal component analysis (PCA). The PCA result (Figure 2A) showed that tempe was separated into three main groups. The first two groups were samples processed at Gunung Kidul and Ngawi, clustered separately from the tempe processed in Bogor regardless of the fermentation places. This result suggested that the metabolite profiles of tempe were influenced more by where processing happens during PFF rather than FF. This separation was explained by principal component (PC)1 which was 68.8% of the variability from the 81.6% of the total variance explained.

Among the annotated metabolites, some metabolites were highlighted in the loading plot (Figure 2B) as representative contributing metabolites. Representative metabolites contributing to the separation were mannitol, β-alanine, uridine, uracil, threonine, and tyrosine for PC1 and histamine, uridine, gentiobiose, stearic acid, lyxose, and palmitic acid for PC2. As presented, among the most contributing metabolites is a non-essential amino acid β-alanine. The changes in these metabolites might be caused by environmental condition during PFF that would alter the soaking process hence the final pH and the microbiome [11]. It was reported that the alteration in the microbiome resulted in physical and chemical properties of tempe. The same might also true for FF. The differences in environment condition (i.e., humidity and temperature) during FF might alter the growth of *Rhizopus* spp. The changes in *Rhizopus* spp. growth itself could lead to the changes in protein break down rate [26]. In line with this, here, we found that the relative abundance three amino acids were among the most contributing metabolites to the separation.

The temperature and humidity inside the fermentation room during fermentation are environmental factors commonly monitored in the bigger scale industries. Previous study [18] has suggested that these two factors might affect the clustering of tempe based on region of origin According to our observation (Table S1), temperature may not be a dominant factor related to clustering of samples. However, Gunung Kidul samples were distinctively clustered along PC1 and the humidity was notably higher than the other two samples. This finding suggested that humidity during PFF may have a greater influence on tempe metabolite profiles compared to average temperature.

### 2.2. The Soybean, Water and Starter Cultures Affected Metabolite Profiles of Tempe

**Figure 3:**
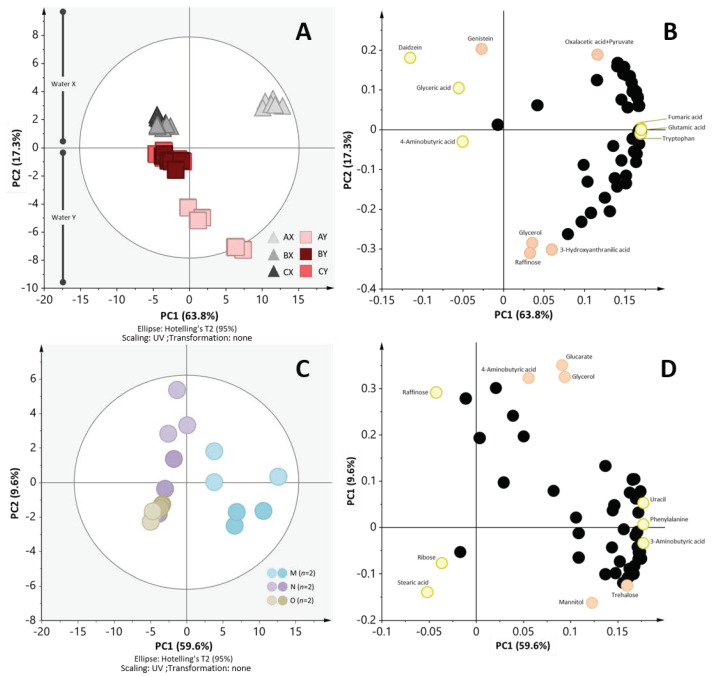
Effect of raw materials and starter culture on metabolite profiles of tempe. (**A**) PCA score scatter plot of tempe made with different water and soybean from 53 annotated metabolites. In this experiment three soybeans were used (A, B, and C; coded by color). In addition, two different water types were used (water X and Y; coded by triangle). In this plot, two biological replicates and three chemical replicates from each sample are presented. Here, it shows that tempe AX and AY are separated from tempe BX, BY, CX, and CY along PC1 (explains 63.8% of variance). Additionally, it is shown that tempe made by water X (triangle) and water Y (rectangular) separated from each other along PC2 (explains 17.3% of variance). (**B**) The PCA loading plot raw material experiment. Representative metabolites are notated in this plot (yellow = PC1; orange = PC2). (**C**) PCA score plot of starter culture set. This figure shows tempe M (blue) has a different metabolite profile to that of tempe N (purple) and O (green) based on PC1 which explains 59.6% of variance. (**D**) The PCA loading plot showing representative metabolites that contribute to separation (yellow = PC1; orange = PC2).

The second experiment aimed to evaluate the effect of soybean and water on the metabolite profiles of tempe. Here we used three types of soybean namely soybeans A, B, and C and two types of water coded water X and Y (Table 1, Table S6, Figure S1). Figure 3A shows the PCA score scatter plot constructed by treating annotated 53 metabolites as the explanatory variables. This figure shows that the clustering of AX and AY were different from the clustering of BX, BY, CX, and CY according to PC1 (63.8% of explained variability). As we have observed, PC1 separated samples according to soybean type, therefore we could conclude that the variability of samples was more affected by the type of soybean although clustering based on PC2 was influenced by the water type (17.3% of the variability). In our initial experiments, we also confirmed that the soybean used in this study had differing metabolite profiles (Figure S2) and therefore, it is reasonable that tempe made from different soybeans resulted in different metabolite profiles.

Daidzein, glyceric acid, 4-aminobutyric acid, fumaric acid, glutamic acid, and tryptophan were some of the metabolites that contributed the most to the separation tempe made with soybean A from soybeans B and C. While genistein, daidzein, oxaloacetic acid+pyruvate, raffinose, hydroxyloanthranilic acid, and glycerol contributed to the separation of tempe made with water X and water Y. Here we observed that metabolites that have been reported to show health benefits (antioxidant, radical scavenging activity) [3] such as isoflavone daidzein, genistein, and amino acids (e.g., glutamic acid and tryptophan) were among the most affected metabolites. Therefore, it is suggested that the soybean and water of choice might be crucial factors to be considered by the industries which aim to take health benefits of tempe (i.e., production of isoflavone isolates, protein isolates, etc.). The complete list of metabolites is provided in Table S2. 

### 2.3. Different Brands of Commercial Starter Culture of Tempe Affect Metabolite Profiles of Tempe

In Indonesia, commercial starter is commonly use in both small and bigger scale tempe industries. For this reason, we used commercial starters to test our hypothesis rather than using pure culture of *Rhizopus oligosporus* (*R. oligosporus*) which is commonly used to make lab-scale tempe [1]. There are several brands available in the market although each claiming to contain *R. oligosporus*. Here we used three different brands i.e., M, N, and O. These brands are the most readily available in Indonesian online marketplace while the starter N is known to be the most popular among the three. Since our objectives are not to directly compare the activity of these starter cultures, disclosure of all brand names is not necessary.

Upon PCA analysis (Figure 3C), there was a clear separation between tempe made with starter culture M and tempe made with starter cultures N and O which are clustered together. Here we note that samples N and O were also separated by PC2 to some extent with 9.7% explained variable. Through this experiment, we could get some insights on how different brands of commercial *R. oligosporus* would also affect the metabolite profiles of tempe. Therefore, the brand of starter culture could be one of the important factors in tempe production.

Previous researches have attempted to compare the characteristics of tempe made using different pure culture of *R. oligosporus* strains. Despite being inoculated as a pure culture, tempe made with different *R. oligosporus* strains showed only slightly different characteristics [19]. Therefore, we argue that the difference in metabolite profiles found in our study may not be due to different strain use in the commercial culture and or the microbial profile. Rather, we suggested a hypothesis that this separation might be due to unknown and unstandardized initial cell numbers in each starter culture brand. However, further analysis will be needed to prove this hypothesis.

All commercial cultures used in our study were prepared in powdered form in an unsterile package (Figure S1). There is very limited to no information provided by the manufacturer of the starter culture on what strain is used in the starter culture and how the product was produced. Most importantly, there is no information that specifies the ingredients or initial cell count in all brands. Nonetheless, we can generally draw an insight that it is important for the artisan and/or tempe industry to stick to one starter culture brand in order to maintain the quality of their product.

Based on the PCA loading plot, stearic acid, raffinose, ribose, uracil, phenylalanine, and 3-aminobutyric acid are among the metabolites that contribute the most to the separation. It is interesting to find raffinose among the most contributing metabolites while this metabolite is usually referred to as an anti-nutrient [3]. Raffinone has been known to cause flatulence associated with bean-based foods. Further study on this issue might give interesting insights to improve nutritive value of tempe. The complete list of metabolites is provided in Table S2.

### 2.4. Japanese Tempe and Indonesian Tempe were Clustered Separately in the Validation Set 

According to the first and the second experiment, we could conclude the generality that the end-product of tempe was affected by the processing, soybean, water, and the starter culture. However, which aspects affect the metabolite profiles more were still unclear. Therefore, we collected unbiased industrial samples from Indonesia and Japan to validate our hypothesis. Eight samples from Japanese industries and seven samples from Indonesian industries were collected and subjected to GC/MS analysis. Upon analysis, 53 metabolites were annotated. Based on these metabolites, the PCA results show a general separation between Indonesian tempe and Japanese tempe (Figure 3A). PC1 explained 32.8% of the variance while PC2 explained 20.7% of the variance.

The separation between Indonesian tempe and Japanese tempe can be observed. In the PCA, tempe JP8 was clustered together with tempe ID2 and tempe ID4 which were obtained from Indonesia under general clustering of Indonesian and Japenese tempe.

**Figure 4:**
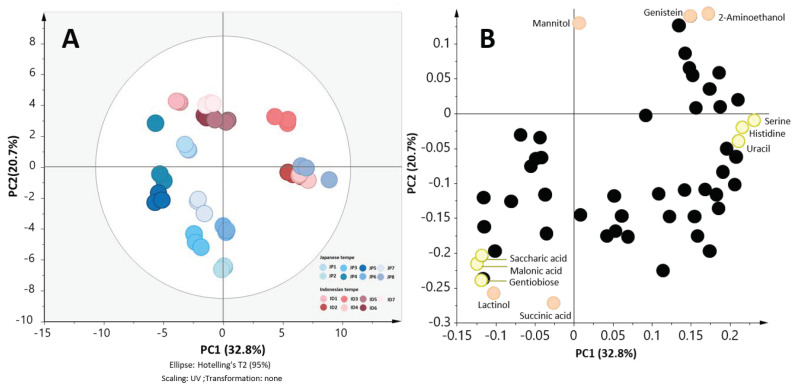
The PCA score and loading of the validation set. (**A**) This figure shows PCA score scatter plot of metabolite profiles of eight Japanese industries (coded JP1-JP8) and seven Indonesian industries (coded ID1-ID7). Along PC2, tempe samples were clustered based on the origin except for JP8. (**B**) Some of the most contributing metabolites to this separation were mannitol, genistein, 2-aminoethanol, succinic acid, lactinol, and gentiobiose.

The loading plot of the validation set (Figure 4B) showed that Indonesian tempe which visually clustered at the positive quadrant of PC1 and PC2 were higher in almost all amino acids except for isocitric+citric acid. Furthermore, the relative abundance of detected isoflavone (daidzein and genistein) were also higher in Indonesian tempe. However, Japanese tempe were higher in some organic acids including malonic acid, saccharic acid, as well as lactic acid. 

The clustering of tempe JP8 as a Japanese tempe with Indonesian tempe implied that although the sample was made using obviously different soybean, water, and completely different environment conditions throughout the production, there are other factors that lead to this clustering. Furthermore, upon confirmation via personal communication, we learned that tempe JP8 optimized its own production method, which were distinct from common methods used in Indonesian or Japanese industries. Interestingly, it was also stated that tempe JP8 was made by using Indonesian commercial starter culture (starter N, which was used in this study).

Based on such information, we can suggest an insight that starter culture might play bigger a role than other tested factors. It is important to note that other Japanese industries other than industry tempe JP8 refused to disclose such detailed information on their production methods. Therefore, further investigation on the influence of starter cultures to tempe metabolite profile must be conducted in order to verify the findings from this study.

### 2.5. Conclusions

The production of tempe is not a natural fermentation. Most of the production steps include man-driven processes such as washing, boiling, as well as inoculation of the starter culture.

To date, the production of tempe is not strictly controlled although the standardization documents are available. This is related to the fact that tempe is a low-cost food produced by small to middle scale producers, therefore, sophisticated control is not feasible to be applied in smaller or middle scale tempe producers.

Tempe has been gaining popularity throughout the world for its unique taste and health benefits (i.e., plant-based protein source). Although many studies have been done to understand and improve tempe fermentation, a few have been focusing on a rather broader view of tempe production in the industry. This leads to fragmented information that is rather far from the application.

In the production of tempe, there are factors that will highly depend on where and how tempe is made. For instance, although there is general suggestion that soybean that is used for tempe production should be of good quality, it is almost impractical to enforce the usage of certain varieties or genotypes only to all tempe producers all over the world.

The availability of raw materials (water, soybean, and starter cultures) and the uncontrollable factors (climate, ground water quality, indigenous microbes, etc.) should be taken into consideration when larger industries aim to produce tempe for more delicate purposes such as the production of tempe for pharmaceutical products and functional food. On the other hand, such careful quality control will be too laborious and cost ineffective for common tempe producers. Therefore, this study aim to propose comprehensive information as a basis for the industry to control their production.

Through this study, we provide general insight on how environmental condition, water type/quality, soybean genotype, and starter culture would affect the quality of tempe as seen through the metabolite profiles. Humidity of the production region affects metabolite profile more than temperature. Soybean and water affected metabolite profiles of tempe. It was shown that the influence of soybean was more than the influence of water. Starter cultures influenced metabolite profiles of tempe. Based on the validation set, it was suggested that the starter culture might affect metabolite profiles more than other tested factors.

This information might be useful for the larger scale industries and/or policy makers to ensure the quality of tempe for the benefit of the consumers. For smaller scale producers, this knowledge will be beneficial for improvement of the existing procedure.

## 3. Materials and Methods

### 3.1. Tempe Production in Laboratory

In this research three different soybeans were used (A, B and C) (Table S6). These soybeans were purchased from the online marketplace. Two different water sources (X and Y) were used to represent different types of water used. Water X was laboratory grade pure water (pH 9.8) while water Y was bottled drinking water (pH 8.7). Using these ingredients, tempe were made inside the laboratory environment according to the method shown in Figure 4. Unique codes were used to identify the soybean-water and soybean-starter combinations (Table 1). After fermentation, tempe samples were quenched by rapid freezing method (10 min in liquid nitrogen), grounded into fine powder by using microbead shockers, then lyophilized overnight to remove water content [18]. The powdered tempe was kept in −30 °C until analysis.

### 3.2. Cross Fermentation Experiment

This experiment was done to reveal the effect of uncontrolled fermentation environment on the metabolite profile of tempe. We collaborated with three middle-range industries located in three different cities in Indonesia namely Bogor, Ngawi, and Gunung Kidul (Figure S3). These industries apply identical tempe production steps. The main idea of this experiment is to undergo PFF and FF of tempe (Figure 1) at two different cities.

Cross fermentation allowed us to separately focus on the effect of factors related to place of PFF (water, soybean, and starter in use) and place of FF (environmental condition during fungal fermentation). This experiment was completed as follows: firstly, tempe underwent PFF at one city (for example: Bogor). After inoculation of starter culture and packing, tempe was taken to other collaborating industries to undergo FF (for example, at Ngawi or at Gunung Kidul). Sample codes are listed in Table 1. Temperature and humidity of fermentation room were measured (Tanita, Tokyo, Japan).

### 3.3. Validation Sample Collection

Eight samples were bought from Japanese industries through online marketplaces while seven industrial samples were brought from Indonesia to Japan in an ice-chilled insulated bag. Indonesian tempe samples were obtained from six different cities in three different islands in Indonesia (Table S2). Upon arrival in Japan, all Indonesian samples were quenched by rapid-freezing method in liquid nitrogen then were grounded into fine powder by using microbead shockers. Similar treatment was given to all tempe samples bought in Japan. The powdered tempe was kept in −30 °C until analysis. Sample details are provided in supplementary materials.

### 3.4. Sample Sets

In this research, we classified the samples into several groups and sets. The first group was a sample group to investigate the effect of raw materials, namely: soybean set, water set, starter-culture set. The second group was the validation group that consisted of industrial samples mentioned in Section 3.3.

### 3.5. Extraction and Derivatization

Extraction solvent (methanol:chloroform:ultrapure water = 5:2:2) containing internal standard (ribitol 0.1 mg/mL) was added to 15 mg tempe powder. Blank samples were prepared by adding the mixed solvent into empty microtubes. These blanks were treated equally throughout the extraction just as normal samples. Subsequently, all samples were mixed for 30 min at 1200 rpm (37 °C) followed by centrifugation for 3 min at 4 °C (10,000× *g*). The aqueous supernatant (400 µL) was transferred to a new 1.5 mL microtube. Then, ultrapure water (400 µL) was added before followed by 1-min mixing. Centrifugation was done afterwards to separate hydrophilic and hydrophobic fractions. The upper phase (400 µL) was transferred into new tubes. The rest of the upper phase from each sample, including blank, was collected together to make a quality control (QC) pool then 400 µL samples from QC pool were transferred to a new tube. All samples and QCs were subjected to evaporation (using centrifuge concentrator, 25 °C). After around 50% of the solvent was evaporated, samples were lyophilized overnight.

Oximation and silylation protocols were used to derivatize the metabolites before GC/MS analysis. Oximation was done to improve volatility of compounds with carboxyl groups It was carried out by adding 100 µL Methoxyamine hydrochloride (20 mg/mL in pyridine) (Sigma-Aldrich, St. Louis, MO, USA) into lyophilized samples then incubated in a shaker incubator (30 °C, 90 min, 1200 rpm). After oximation, silylation was conducted. Silylation is one of common derivatization methods to improve volatility of compounds containing acidic hydrogen groups (hydroxyl, carboxylic acid, amine, thiol, phosphate). Silylation was done by adding 50 μL of *N*-Methyl-*N*-trimethylsilyl-trifluoroacetamide (MSTFA) (GL Science, Tokyo, Japan) to the mixture. Mixtures were then subjected to the second incubation (37 °C, 30 min, 1200 rpm).

### 3.6. GC/MS-Based Metabolite Profiling

The analysis was done separately for each sample set. We utilized GC/MS-QP2010 Ultra (Shimadzu, Kyoto, Japan) equipped with Inert Cap 5MS/NP column (GL Science, Tokyo, Japan) and auto-sampler AOC-20i/s (Shimadzu, Kyoto, Japan). Machine conditions were adjusted similarly to the previously reported research [18]. For each analysis, a standard alkane mixture (C8-C40) was injected then the retention time data of each peak in the alkane mixture was used as a reference of retention index (RI) for tentative identification.

### 3.7. Metabolite Annotation and Statistical Analysis

Raw data from GC/MS analysis were firstly converted from GCMSsolution MS data files (.QDC) to ABF (Analysis Base Format) using downloadable freeware Reifycs Abf Converter (https://www.reifycs.com/AbfConverter/, Reifycs, Tokyo, Japan). Peak alignment, annotation, and filtering were carried out in MS-DIAL (Tsugawa, H, 2015) (http://prime.psc.riken.jp/compms/msdial/main.html, RIKEN, Tokyo, Japan). Tentative annotation of metabolites was done based recorded retention index (RI) on recorded RI GL-Science DB (InertCap 5MS-NP, predicted Fiehn RI, 494 records), downloadable on MS-DIAL official website. In the annotation step, the QC sample acted as reference. Metabolite peaks were considered if the height shows five times higher intensity than the blank (see supplementary materials). Furthermore, additional filtering was applied by selecting metabolites which show relative standard deviation (RSD) less than 30% within QC samples. Tentatively annotated metabolites were subjected to principal component analysis (PCA) by using SIMCA P+ ver. 13.0.3 package (Umetrics, Umea, Sweden).

## Figures and Tables

**Table 1:** Sample codes list.

Data Set	Code	Denotation
Cross fermentation	BB	PFF in Bogor, FF in Bogor
	BK	PFF in Bogor, FF in Gunung Kidul
	BG	PFF in Bogor, FF in Ngawi
	GB	PFF in Ngawi, FF in Bogor
	KB	PFF in Gunung Kidul, FF in Bogor
Soybean-water effect	AX	Soybean A, water X
	BX	Soybean B, water X
	CX	Soybean C, water X
	AY	Soybean A, water Y
	BY	Soybean B, water Y
	CY	Soybean C, water Y
Starter culture effect	M	Starter culture M
	N	Starter culture N
	O	Starter culture O

PFF = pre fungal fermentation; FF = fungal fermentation.

## Supplementary Materials

The following are available online at https://www.mdpi.com/2218-1989/10/9/367/s1, Table S1: Average of temperature and humidity during cross fermentation experiment, Table S2: Sample codes of the validation set, Table S3: Annotated metabolite list of cross fermentation experiment, Table S5: Annotated metabolite list of raw materials experiment, Table S6: Annotated metabolites of starter cultures experiment, Table S7: Soybean in use, Figure S1: Starter culture in use, Figure S2: PCA score plot of three different soybeans; Figure S3: Cross fermentation scheme.
