# Supplementary material for: Metabolomics-Based Study of the Effect of Raw Materials to the End Product of Tempe—An Indonesian Fermented Soybean
Source: Metabolites. 2020 Sep 11;10(9):367. doi: 10.3390/metabo10090367 (PMC7569771; doi:10.3390/metabo10090367)

metabolites

MDPI

Supplementary materials

Table S1. Average of temperature and humidity during cross fermentation experiment

City	*Average Temperature (ºC)	*Average Humidity (%)
Bogor	29.9	59.9
Gunung Kidul	29.2	64.6
Ngawi	27.9	61.0

*Average of temperature and humidity during production

Table S2. Sample code of the validation set

Sample code	Country of origin	City of origin	Starter culture
ID1	Indonesia	Bogor	Starter N
ID2	Indonesia	Bandung	Starter N
ID3	Indonesia	Semarang	Starter N
ID4	Indonesia	Banjarbaru	Starter N
ID5	Indonesia	Pekanbaru	Starter N
ID6	Indonesia	Banjarbaru	Starter N
ID7	Indonesia	Ngawi	Starter N
JP1	Japan	Okayama	Unknown
JP2	Japan	Nagano	Unknown
JP3	Japan	Akita	Unknown
JP4	Japan	Akita	Unknown
JP5	Japan	Tokyo	Unknown
JP6	Japan	Niigata	Unknown
JP7	Japan	Okayama	Unknown
JP8	Japan	Otsu	Starter N

Table S3 Annotated metabolite list of cross fermentation experiment

No	Metabolite	Average RI*	Average Quantitative m/z	Annotation
1	2-Aminoadipic acid	1725.08	260.0975	GL-sciences library
2	2-Aminobutyric acid	1177.32	130.125	GL-sciences library
3	2-Aminoethanol	1275.19	174.1	GL-sciences library
4	3-Hydroxy-3-methylglutarate	1612.48	147.1031	GL-sciences library
5	3-Hydroxyanthranilic acid	1885.68	264.0882	GL-sciences library
6	3-Phenyllactic acid	1598.4	193.0633	GL-sciences library
7	4-Aminobutyric acid	1541.29	174.1	GL-sciences library
8	4-Hydroxyphenethyl alcohol	1580.02	179.0725	GL-sciences library
9	Alanine_2TMS	1106.28	116.1	GL-sciences library
10	Asparagine_3TMS	1682.53	116.1075	GL-sciences library
11	Aspartic acid_3TMS	1529.67	232.0625	GL-sciences library
12	b-Alanine_3TMS	1436.5	174.0945	GL-sciences library
13	Cadaverine	1856.57	174.095	GL-sciences library
14	Fumaric acid	1346.95	245.0177	GL-sciences library
15	Galactitol	1978.4	217.05	GL-sciences library
16	Galactose_1	1925.05	319.1	GL-sciences library
17	Galactose_2+Glucose_2	1951.12	319.0833	GL-sciences library
18	Gentiobiose_2_minor	2916.02	361.1175	GL-sciences library
19	Glucarate_3_same	2059.48	333.09	GL-sciences library
20	Glucose_1	1931.17	319.1028	GL-sciences library
21	Glutamic acid_3TMS	1628.13	246.0813	GL-sciences library
22	Glutamine_3TMS	1784.88	156.095	GL-sciences library
23	Glyceric acid	1338.79	189.0455	GL-sciences library
24	Glycerol	1280.54	147.1	GL-sciences library
25	Glycine_3TMS	1316.32	174.1	GL-sciences library
26	Glycolic acid	1074.69	147.15	GL-sciences library
27	Histamine	1890.28	174.1	GL-sciences library
28	Histidine_3TMS	1940.52	154.1	GL-sciences library
29	Hydroxyproline	1538.15	230.0933	GL-sciences library
30	Hypoxanthine	1820.72	265.0471	GL-sciences library
31	Inositol	2131.89	217.0575	GL-sciences library
32	Isocitric acid+Citric acid	1838.03	273.0667	GL-sciences library
33	Isoleucine_2TMS_major	1300.76	158.1475	GL-sciences library
34	Lactic acid	1063.17	147.1	GL-sciences library
35	Leucine_2TMS	1278.72	158.15	GL-sciences library
36	Lysine_4TMS	1938.62	174.0975	GL-sciences library
37	Lyxose_2_major	1683.5	103.0591	GL-sciences library

38	Malic acid	1496.06	147.1	GL-sciences library
39	Malonic acid	1206.88	147.1025	GL-sciences library
40	Mannitol	1966.02	319.1059	GL-sciences library
41	Mannose_1_major	1917.93	319.105	GL-sciences library
42	Melibiose_1_major	2938.44	204.05	GL-sciences library
43	Meso erythritol	1521.74	174.0952	GL-sciences library
44	n-Butylamine	1112.25	174.0905	GL-sciences library
45	Nicotinic acid	1297.54	180.0111	GL-sciences library
46	Oxalacetic acid+Pyruvate	1050.03	174.0361	GL-sciences library
47	Oxalate	1133.01	147.0912	GL-sciences library
48	Phenylalanine_2TMS	1642.61	218.05	GL-sciences library
49	Phosphate	1281.37	299.0316	GL-sciences library
50	Plamitic acid(16:0)	2046.12	117.055	GL-sciences library
51	Proline_2TMS	1306.07	142.145	GL-sciences library
52	Propyleneglycol	1000.64	117.0867	GL-sciences library
53	Putrescine	1757.66	174.1025	GL-sciences library
54	Pyroglutamic acid_2TMS	1534.33	156.09	GL-sciences library
55	Ribose	1698.67	103.0525	GL-sciences library
56	Serine_3TMS_major	1368.92	204.07	GL-sciences library
57	Stearic acid(17:0)	2243.84	117.0525	GL-sciences library
58	Succinic acid(or anhydride)	1316.23	147.1	GL-sciences library
59	Threonine_3TMS_major	1396.85	218.095	GL-sciences library
60	Thymine	1410.12	255.0632	GL-sciences library
61	Tryptamine_1	2232.89	128.1	GL-sciences library
62	Tryptophan_3TMS	2250.84	202.0533	GL-sciences library
63	Tyrosine	1957.72	218.0575	GL-sciences library
64	Uracil	1346.18	241.0225	GL-sciences library
65	Urea	1236.19	147.0917	GL-sciences library
66	Uric acid	2124.92	441.0923	GL-sciences library
67	Uridine_4TMS	2478.34	217.0467	GL-sciences library
68	Valine_2TMS	1222.66	144.1475	GL-sciences library
69	Xanthine	2039.23	353.1	GL-sciences library
70	Xylitol	1732.13	217.0553	GL-sciences library
71	Xylonic acid	1793.14	292.0833	GL-sciences library

Note: Listed alphabetically

Table S4. Annotated metabolite list of raw materials experiment

No	Metabolite	Average RI*	Average Quantitative m/z	Annotation
1	1,3-Propanediamine	1666.48	174.092	GL-sciences library
2	2-Aminoadipic acid	1726.65	260.1	GL-sciences library
3	2-Aminobutyric acid	1178.32	130.074	GL-sciences library
4	2-Aminoethanol	1275.8	174.1023	GL-sciences library
5	3-Hydroxyanthranilic acid	1886.73	354.11	GL-sciences library
6	4-Aminobutyric acid	1541.79	174.1009	GL-sciences library
7	Arabionose	1684.94	103.0417	GL-sciences library
8	Arabitol	1746.3	217.0917	GL-sciences library
9	Asparagine_3TMS	1683.67	116.0554	GL-sciences library
10	Aspartic acid_3TMS	1531.06	232.0706	GL-sciences library
11	b-Alanine_3TMS	1437.11	174.1016	GL-sciences library
12	b-Lactose_2_minor	2780.04	204.075	GL-sciences library
13	Cadaverine	1856.44	174.1091	GL-sciences library
14	Daidzein	2974.34	398.0932	GL-sciences library
15	Fumaric acid	1348.55	245.0331	GL-sciences library
16	Galactitol	1980.03	217.0661	GL-sciences library
17	Genistein	2989.14	471.1532	GL-sciences library
18	Gentiobiose_2_minor	2915.96	204.0839	GL-sciences library
19	Glucuronate_1_major	1975.09	333.1	GL-sciences library
20	Glutamic acid_3TMS	1629.82	246.1011	GL-sciences library
21	Glutamine_3TMS	1785.93	156.0861	GL-sciences library
22	Glyceric acid	1340.38	147.0554	GL-sciences library
23	Glycerol	1282.24	147.05	GL-sciences library
24	Glycine_3TMS	1316.77	147.0598	GL-sciences library
25	Histidine_3TMS	1941.2	154.0847	GL-sciences library
26	Hydroxyproline	1539.13	230.0917	GL-sciences library
27	Hypoxanthine	1820.75	265.0618	GL-sciences library
28	Inositol	2132.41	217.0786	GL-sciences library
29	Isocitric acid+Citric acid	1839.83	273.0627	GL-sciences library
30	Isoleucine_2TMS_major	1301.89	158.1276	GL-sciences library
31	Lactic acid	1064.22	147.05	GL-sciences library
32	Leucine_2TMS	1279.73	158.1155	GL-sciences library
33	Lysine_4TMS	1939.86	174.1017	GL-sciences library
34	Malic acid	1497.78	147.0524	GL-sciences library
35	Malonic acid	1208.02	147.0588	GL-sciences library
36	Meso erythritol	1523.84	217.08	GL-sciences library
37	Methionine_2TMS	1530.53	176.0859	GL-sciences library
38	Oxalacetic acid+Pyruvate	1051.09	174.0508	GL-sciences library
39	Phenylalanine_2TMS	1642.66	218.0722	GL-sciences library
40	Proline_2TMS	1306.53	142.0917	GL-sciences library
41	Propyleneglycol	1002.93	117.0193	GL-sciences library
42	Putrescine	1757.66	174.11	GL-sciences library
43	Raffinose	3501.76	361.1537	GL-sciences library

44	Saccharopine_1_major	2474.42	357.1558	GL-sciences library
45	Serine	1370.3	204.0992	GL-sciences library
46	Threonine	1301.37	117.025	GL-sciences library
47	Thymine	1410.57	255.0516	GL-sciences library
48	Tryptophane	2218.76	218.08	GL-sciences library
49	Tyramine	1932.76	174.1	GL-sciences library
50	Uric acid	2126.88	441.1653	GL-sciences library
51	Valine_2TMS	1223.53	144.1047	GL-sciences library
52	Xanthine	2042.52	205	GL-sciences library
53	Xylitol	1733.91	217.084	GL-sciences library

Note: Listed alphabetically

Table S5 Annotated metabolite list of starter culture experiment

No	Metabolite	Average RI*	Average Quantitative m/z	Annotation
1	2-Aminoadipic acid	1726.48	260.1167	GL-sciences library
2	2-Aminobutyric acid	1178.48	130.0667	GL-sciences library
3	2-Aminoethanol	1275.79	174.0913	GL-sciences library
4	3-Hydroxy-3-methylglutarate	1613.65	247.1046	GL-sciences library
5	3-Hydroxyanthranilic acid	1886.61	354.0792	GL-sciences library
6	4-Aminobutyric acid	1541.79	174.0842	GL-sciences library
7	Alanine_3TMS	1371.92	188.0978	GL-sciences library
8	Asparagine_3TMS	1683.52	116.0457	GL-sciences library
9	Aspartic acid_3TMS	1531.12	232.0605	GL-sciences library
10	b-Alanine_3TMS	1437.04	174.0935	GL-sciences library
11	b-Lactose_2_minor	2779.55	147.1	GL-sciences library
12	Fumaric acid	1348.48	245.025	GL-sciences library
13	Galactose_1	1926.61	319.0917	GL-sciences library
14	Galactose_2+Glucose_2	1952.87	319.095	GL-sciences library
15	Glucarate_3_same	2059.6	333.0643	GL-sciences library
16	Glutamic acid_3TMS	1629.72	246.0952	GL-sciences library
17	Glutamine_3TMS	1785.83	156.0725	GL-sciences library
18	Glycerol	1282.34	147.0455	GL-sciences library
19	Glycine_3TMS	1316.73	174.0929	GL-sciences library
20	Histidine_3TMS	1941.15	154.075	GL-sciences library
21	Inositol (add)	2132.31	217.0674	GL-sciences library
22	Isocitric acid+Citric acid	1839.75	273.0604	GL-sciences library
23	Isoleucine_2TMS_major	1301.74	158.15	GL-sciences library
24	Lactic acid (add)	1064.61	117.01	GL-sciences library
25	Leucine_2TMS	1279.72	158.11	GL-sciences library
26	Lysine_4TMS	1939.72	174.0957	GL-sciences library
27	Lyxose_2_major	1685.25	103.0133	GL-sciences library
28	Malic acid	1497.74	147.0458	GL-sciences library
29	Malonic acid	1208.04	147.025	GL-sciences library
30	Mannitol	1967.44	319.1278	GL-sciences library
31	Methionine_2TMS	1530.59	176.0881	GL-sciences library
32	Nonanoric acid(9:0)	1367.46	116.9625	GL-sciences library
33	n-Propylamine	1027.74	174.0979	GL-sciences library
34	Oxalacetic acid+Pyruvate	1051.54	174.0196	GL-sciences library
35	Phenylalanine_2TMS	1642.52	218.0667	GL-sciences library
36	Phosphate	1282.5	299.0125	GL-sciences library
37	Palmitic acid (16:0)	2047.16	132.05	GL-sciences library
38	Proline_2TMS	1303.43	142.05	GL-sciences library

39	Propyleneglycol	1003.2	117.0043	GL-sciences library
40	Pyroglutamic acid_2TMS	1534.42	156.0568	GL-sciences library
41	Raffinose	3500.73	361.1056	GL-sciences library
42	Ribose	1752.17	217.05	GL-sciences library
43	Serine_3TMS_major	1371.77	189.1	GL-sciences library
44	Stearic acid(17:0)	2244.83	116.9913	GL-sciences library
45	Succinic acid(or anhydride)	1316.93	147.05	GL-sciences library
46	Sucrose	2703.69	361.0555	GL-sciences library
47	Threonine_3TMS_major	1397.94	218.1318	GL-sciences library
48	Trehalose	2815.43	361.1167	GL-sciences library
49	Tryptophan_3TMS	2249.96	202.075	GL-sciences library
50	Tyrosine	1958.29	218.0625	GL-sciences library
51	Uracil	1346.73	241.0413	GL-sciences library
52	Urea	1238.53	147.0412	GL-sciences library
53	Valine_2TMS	1223.5	144.0958	GL-sciences library
54	Xanthine	2040.07	353.0886	GL-sciences library

Note: Listed alphabetically

Table S6 Soybeans in use

Group	Picture	Code	Source
**Soybean**	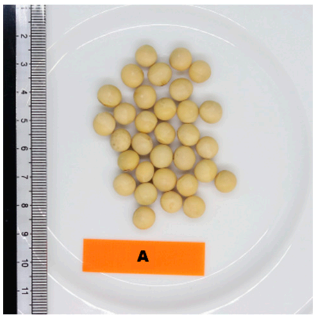	**A**	Japanese market place
	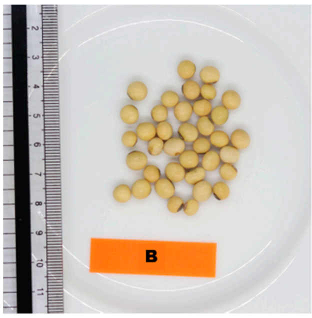	**B**	Indonesian market place
	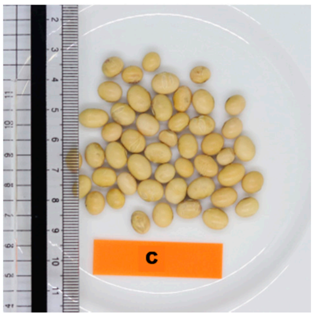	**C**	Indonesian market place

Figure S1 Commercial starter in use

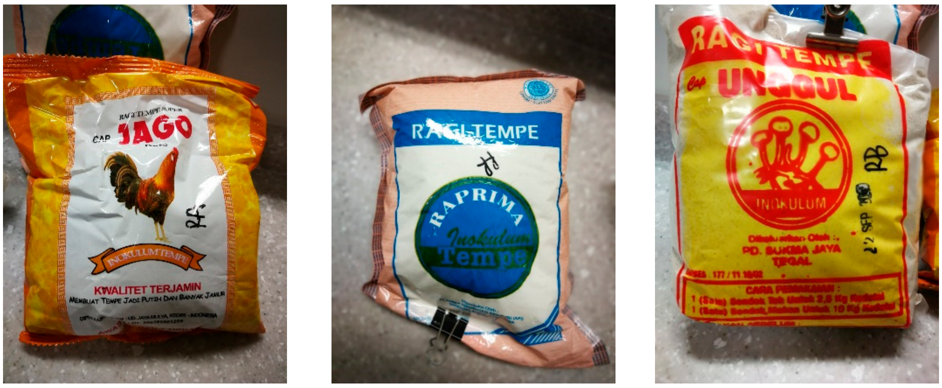

Starter M Starter N Starter O

Figure S2 PCA score plot of three different soybean

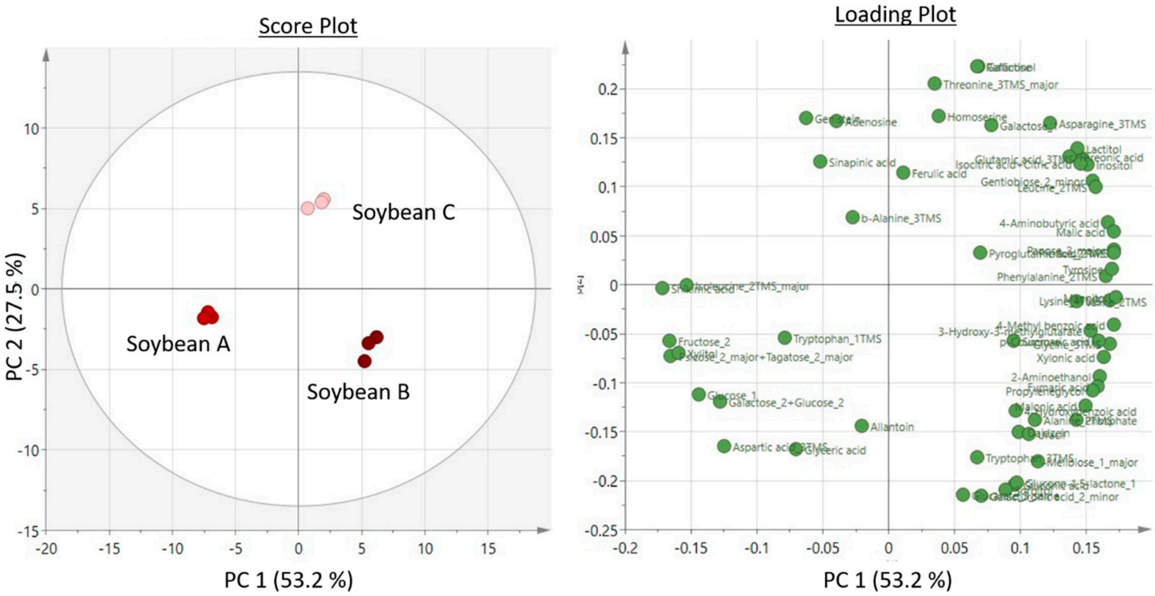

Indonesian imported soybean , Indonesia & Japan have different metabolites profile

Figure S3 Cross fermentation scheme

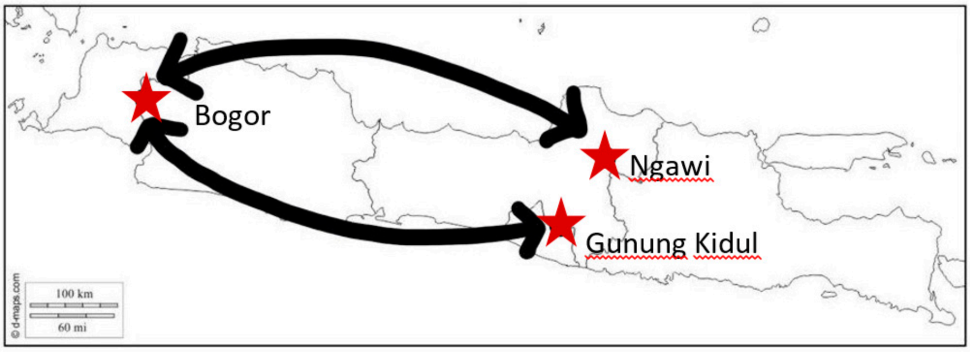

Supplement: Supplementary file 1 [file metabolites-10-00367-s001.pdf]
